# Bone marrow macrophage‐derived exosomal miR‐143‐5p contributes to insulin resistance in hepatocytes by repressing MKP5

**DOI:** 10.1111/cpr.13140

**Published:** 2021-10-14

**Authors:** Linfang Li, Huiyan Zuo, Xiuqing Huang, Tao Shen, Weiqing Tang, Xiaoyi Zhang, Tong An, Lin Dou, Jian Li

**Affiliations:** ^1^ The Key Laboratory of Geriatrics Beijing Institute of Geriatrics Beijing Hospital, National Center of Gerontology National Health Commission; Institute of Geriatric Medicine Chinese Academy of Medical Sciences Beijing China; ^2^ Graduate School of Peking Union Medical College Beijing China

**Keywords:** bone marrow macrophage, exosome, hepatic insulin resistance, miR‐143‐5p, MKP5

## Abstract

**Objective:**

In this study, we aim to explore the role of bone marrow macrophage‐derived exosomes in hepatic insulin resistance, investigate the substance in exosomes that regulates hepatic insulin signalling pathways, reveal the specific molecular mechanisms involved in hepatic insulin resistance and further explore the role of exosomes in type 2 diabetes.

**Materials and methods:**

High‐fat diet (HFD)‐fed mice were used as obesity‐induced hepatic insulin resistance model, exosomes were isolated from BMMs which were extracted from HFD‐fed mice by ultracentrifugation. Exosomes were analysed the spectral changes of microRNA expression using a microRNA array. The activation of the insulin signalling pathway and the level of glycogenesis were examined in hepatocytes after transfected with miR‐143‐5p mimics. Luciferase assay and western blot were used to assess the target of miR‐143‐5p.

**Results:**

BMMs from HFD‐fed mice were polarized towards M1, and miR‐143‐5p was significantly upregulated in exosomes of BMMs from HFD‐fed mice. Overexpression of miR‐143‐5p in Hep1‐6 cells led to decreased phosphorylation of AKT and GSK and glycogen synthesis. Dual‐luciferase reporter assay and western blot demonstrated that mitogen‐activated protein kinase phosphatase‐5 (*Mkp5*, also known as *Dusp10*) was the target gene of miR‐143‐5p. Moreover, the overexpression of MKP5 could rescue the insulin resistance induced by transfection miR‐143‐5p mimics in Hep1‐6.

**Conclusion:**

Bone marrow macrophage‐derived exosomal miR‐143‐5p induces insulin resistance in hepatocytes through repressing MKP5.

## Graphical abstract

Up‐regulated miR‐143‐5p in BMM exosomes could be passed to hepatocytes and impaired hepatic glycogenesis by targeting *Mkp5*.

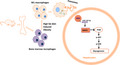

## 1 INTRODUCTION

Insulin resistance is defined as a decrease in the sensitivity and responsiveness of insulin and is the critical pathogenesis of type 2 diabetes.^1^ Decreased glycogenesis is a hallmark of insulin resistance in hepatocytes.^2^ Overweight and obesity are prevalent in the general population,^3^ and obesity has been recognized as a significant risk factor for the development of type 2 diabetes.^4^ Compared with normal BMI, the risk of diabetes is increased by 10–40 times for BMI > 30.^5^ Obesity causes insulin resistance in a variety of ways, and inflammation caused by obesity is an important factor.^6^

Chronic inflammation induced by obesity could lead to hepatic insulin resistance. A significant manifestation of tissue inflammation caused by obesity is the accumulation of macrophages in adipose tissue and the liver.^7^ Activated macrophages are usually divided into two categories: M1 macrophages and M2 macrophages. Both M1 macrophages and M2 macrophages are closely related to inflammatory responses, where M1 macrophages are mainly involved in proinflammatory responses and M2 macrophages are mainly involved in anti‐inflammatory responses.^8^ Studies have found that during obesity, hepatic macrophages towards proinflammatory M1 polarization, which further induces insulin resistance or other liver diseases.^9^ Liver macrophages consist of a resident macrophage population, termed as Kupffer cells, and recruited hepatic macrophages, which infiltrate the liver under obese conditions.^10^ The accumulation of bone marrow‐derived macrophages (BMMs) in adipose tissue is an important cause of insulin resistance in adipose tissue under chronic inflammation resulting from obesity.^11^ The evidence also showed that the infiltration of BMMs in the liver was related to liver damage. The recruitment of BMMs to the liver is regulated by inflammation and fibrosis in the liver.^12^, ^13^ Based on this fact, we performed immunofluorescence staining of macrophages in the liver of HFD‐fed mice and found that there were more macrophages in the liver of HFD‐fed mice compared with chow diet (CD)‐fed mice. Based on the previous studies, we thought that the additional macrophages were recruited from the bone marrow. However, the mechanisms by which BMMs from HFD‐fed mice participate in the regulation of hepatic insulin resistance remain unknown.

Exosomes are vesicle‐like bodies with a diameter of approximately 30–100 nm that are secreted by various types of tissues and cells and play an important role in the pathological and physiological processes in the body.^14^ Exosomes can transmit information in cells by carrying various substances, such as proteins, RNA and lipids.^15^ MicroRNAs are a class of noncoding small RNA molecules with a length of 22 nucleotides. MicroRNAs play an important role in the posttranscriptional regulation of metabolic diseases.^16^ In recent years, many studies have found that microRNAs carried by exosomes play an important role in insulin resistance. An article published in Cell demonstrated that exosomes derived from adipose tissue macrophages could regulate the insulin sensitivity of liver and muscle cells. Further research by this team showed that the miR‐155 carried by exosomes impaired insulin sensitivity.^17^ Similarly, studies also found that adipose tissue‐derived exosomes carrying miR‐27a induce insulin resistance in skeletal muscle by inhibiting PPARγ.^18^ These studies indicated that microRNAs carried by exosomes played an important role in insulin resistance.

Here, we found that BMM‐derived exosomes from HFD‐fed mice were able to induce insulin resistance in the mouse hepatic cell line Hep1‐6. Mechanistically, miR‐143‐5p was significantly increased in the BMM‐derived exosomes of HFD‐fed mice, and MKP5 functions as the downstream target of exosomal miR‐143‐5p in the regulation of insulin resistance. Taken together, our study shows the role of BMM‐derived exosomes from HFD‐fed mice in regulating obesity‐associated insulin resistance. Moreover, miR‐143‐5p in exosomes released by BMMs may be a promising target for treating obesity‐related insulin resistance.

## 2 MATERIALS AND METHODS

### 2.1 Animals and treatments

8‐week‐old male wild‐type (WT) C57BL/6J mice were purchased from SPF Biotechnology Co. Ltd. The mice were separated into two groups (eight mice in each group) and fed a standard chow diet or a high‐fat diet (HFD, 45% kcal from fat from Medicine) for 12 weeks. All mouse procedures were approved by the Ethics and Animal Welfare Committee at College of Life Sciences, Beijing Normal University.

### 2.2 Histological analysis of tissues

The liver specimens were embedded in O.C.T. (Tissue‐Tek) and cut into a thickness of 6 µm. For Oil Red O staining, the slides were incubated with Oil Red O (Solarbio) at 37℃ for 30 min, then used 60% 1,2‐propanediol to wash.^19^ For haematoxylin and eosin (H&E) staining, first, the slides were stained with haematoxylin for 5 min, then washed with 1% ethanol hydrochloride for 3 s. After water washing, the slides were incubated with eosin for 3 min and dehydrated with an alcohol gradient.^20^

### 2.3 Luciferase reporter assay

The 3′‐UTR sequences of MKP5 predicted to interact with miR‐143‐5p were PCR‐cloned and then inserted into the pmirGLO(Sangon). HEK293 cells were cotransfected with pmirGLO, pmirGLO‐MKP5‐3′‐UTR and pmirGLO‐MKP5‐mut and miR‐143‐5p mimics (or negative control) using Lipofectamine 2000 for 48 h. Luciferase reporter assays were performed using the Dual‐Glo luciferase assay system according to standard protocols (Promega).

### 2.4 Cell culture

The mouse hepatic cell line Hep1‐6 and macrophage cell line RAW 264.7 were purchased from the American Type Culture Collection. The cells were cultured in high‐glucose Dulbecco's modified Eagle's medium (DMEM) (Gibco, Grand Island) supplemented with 10% foetal bovine serum (Gibco, Grand Island), 100 units/ml penicillin (Invitrogen), and 0.1 mg/ml streptomycin (Invitrogen) at 37℃ with humidified air and 5% CO_2_.

### 2.5 Isolation and cultivation of mice BMMs

Cells were obtained from the tibia and femur bone marrow of chow diet‐fed and HFD‐fed mice and were cultured in the presence of L‐929 conditioned medium as described in detail previously.^12^ The identification of BMMs is assessed using immunocytochemistry analysis of F4/80 expression.

### 2.6 Oleic acid/palmitic acid (O/P) treatment

The method was performed as previously described.^21^ Oleic acid of 0.25 M (Sigma‐Aldrich) and palmitic acid (Sigma‐Aldrich) were dissolved in 100% ethyl alcohol. Before use, the 0.25 M oleic acid, the palmitic acid stock and 5% BSA were incubated in a 60°C water bath for 10 min. Then, 640 μl of the oleic acid or 320 μl of the palmitic acid stock were added drop‐wise to 20 ml of 5% BSA to obtain 8 mM oleic acid and 4 mM palmitic acid, respectively. Before the experiments commenced, the 8 mM oleic acid and the 4 mM palmitic acid were incubated in a 60°C water bath for 10 min. Equal volumes of oleic acid and palmitic acid were mixed together. A total of 300 μM oleic acid/palmitic acid mixture (2:1, M/M) was used to treat RAW264.7 cells for 24 h.

### 2.7 LPS treatment

LPS was used for M1 polarization. RAW264.7 cells were stimulated with LPS (10 ng/ml) for 24 h.

### 2.8 Exosome purification and characterization

After culturing for 72 h, debris and dead cells in the BMM medium were removed by centrifugation at 2000 × g for 20 min and then filtrated through the 0.2 μm filter. The medium was then subjected to ultracentrifugation at 100,000 × g for 4 h at 4°C. After washing with PBS (100,000 × g for 20 min), the exosome‐containing pellet was resuspended in PBS.^17^ The characterization of exosomes was confirmed by measuring the expression of exosome‐specific markers FLOT1, CD63 and CD9 by western blot analysis.

### 2.9 Exosomes tracing

To monitor the exosome trafficking, exosomes were labelled with PKH26 fluorescent dye using the PKH26 fluorescent cell linker kit (Sigma‐Aldrich). After PKH26 staining, the exosomes were washed in PBS and collected by ultracentrifugation (100,000 × g for 20 min) at 4°C. Finally, PKH26 labelled exosomes were resuspended in PBS.^17^

### 2.10 Electron microscopy

Exosomes obtained by ultracentrifugation were sent to the Central Laboratory of Peking Union Medical College for electron microscope examination.

### 2.11 RNA extraction and real‐time PCR

The total RNA was extracted from liver tissues and Hep1‐6 cells using TRIzol reagent (Invitrogen). Real‐time PCR was performed using IQ5 system (Bio‐Rad). The primers used for reverse transcription and real‐time PCR were listed in Table 1 and Table 2.

**TABLE 1:** The RT‐PCR primer sequence

Gene name	Reverse transcription primer sequence 5′−3′
miR−143‐5p	GTCGTATCCAGTGCAGGGTCCGAGGTATTCGCACTGGATACGACCCAGAG
U6 (mouse)	GTCGTATCCAGTGCAGGGTCCGAGGTATTCGCACTGGATACGACAAATATG

**TABLE 2:** The real‐time PCR primer sequence

Gene name	Forward primer	Reverse primer
U6 (mouse)	GCGCGTCGTGAAGCGTTC	GTGCAGGGTCCGAGGT
*Gapdh* (mouse)	GTCGGTGTGAACGGATTTG	AAGATGGTGATGGGCTTCC
miR−143‐5p	CGGGTGCAGTGCTGCAT	AGTGCAGGGTCCGAGGTATT
*Mkp5*	GGGGACAGACTGAGGTAGCA	GCAAAGAACCCCTGGTATTG
18S	GGAAGGGCACCACCAGGAGT	TGCAGCCCCGGACATCTAAG
*Tnf‐α*	CACAGAAAGCATGATCCGCG	ACTGATGAGAGGGAGGCCAT
*Il−6*	AGCCAGAGTCCTTCAGAGAGA	TGGTCTTGGTCCTTAGCCAC
*Il−10*	CAGAGAAGCATGGCCCAGAA	GCTCCACTGCCTTGCTCTTA
*Cd206*	TTGCACTTTGAGGGAAGCGA	CCTTGCCTGATGCCAGGTTA
Argniase1	TTTTAGGGTTACGGCCGGTG	CCTCGAGGCTGTCCTTTTGA
*Mcp−1*	GTCTGTGCTGACCCCAAGAA	AAGGCATCACAGTCCGAGTC

### 2.12 Cell transfection

The mimics and inhibitors of miR‐143‐5p, MKP5 siRNA and negative control siRNA (NC) were purchased from GenePharma. Mimics, inhibitors, siRNA or miR‐negative control were transfected for 48 h using HiPerFect transfection reagent (Qiagen) according to the manufacturer's protocol.

### 2.13 Protein extraction and western blot analysis

RIPA buffer (Solarbio) was used for protein extraction of liver tissues and Hep1‐6 cells. The blot was incubated with HRP‐conjugated anti‐IgG, followed by the detection with ECL (Millipore). Antibodies against P‐AKT(4060S), AKT(9272S), P‐GSK(5558S) and GSK(27C10) were purchased from CST. Antibodies against F4/80(ab16911) and CD63(ab217345) were obtained from Abcam. Antibodies against GAPDH(60004–1‐lg), β‐actin(20536–1‐AP),CD9(20597–1‐AP), Flotillin‐1(15571–1‐AP) and MKP5(11689–1‐AP) were obtained from Proteintech.

### 2.14 Glycogen content measurement

Glycogen content in cells or livers was analysed using a glycogen assay kit (Biovision), according to the manufacturer's instructions.

### 2.15 Co‐culture assay

After the treatment with O/P, RAW264.7 cells were washed with PBS twice and co‐cultured with Hep1‐6 cells at a ratio of 1:1 using a trans‐well plate (0.4 μm polycarbonate filter, Corning) for 48 h, with Hep1‐6 cells placed in the lower chamber and RAW264.7 cells in the upper chamber. Then harvest Hep1‐6 cells for the next experiments.

### 2.16 Immunofluorescence

The slides were fixed to the samples in 4% paraformaldehyde for 30 min at room temperature. Then, the slides were stained with immunofluorescence as previously described.^22^

### 2.17 Statistical analysis

The data represent the mean ± standard error of the mean (SEM). The two‐tailed unpaired student's t‐test was used to compare the differences of the two groups. The ANOVA multiple comparisons test followed by the Turkey post hoc test were used for comparisons of two more groups. *p* < 0.05 was considered to indicate a statistically significant difference.

## 3 RESULTS

### 3.1 HFD‐fed mice developed obesity and insulin resistance accompanied by increased macrophage infiltration in the liver

**FIGURE 1:**
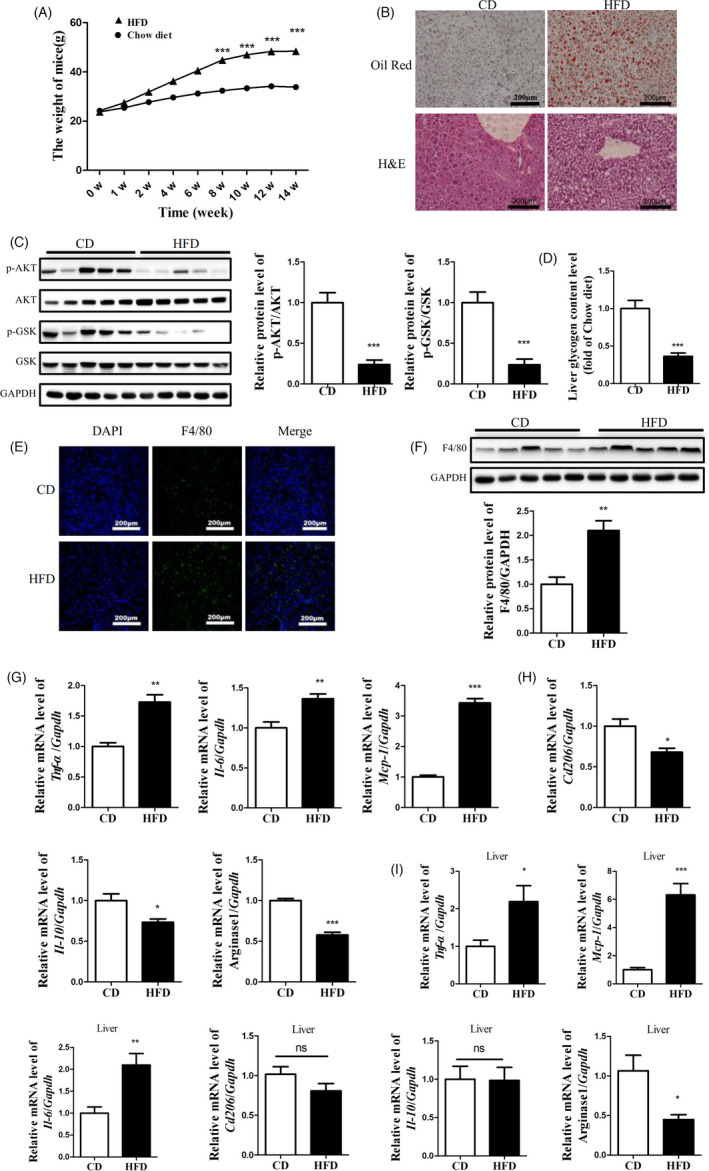
HFD‐fed mice developed obesity and insulin resistance accompanied by increased macrophage infiltration in the liver. (A) The weight of high‐fat diet (HFD)‐fed mice and chow diet (CD)‐fed mice. (B) Oil red O and H&E staining of the livers of the HFD‐fed mice. Relative activation of the AKT/GSK pathway (C) and glycogen level (D) and F4/80 (F) in the livers of HFD‐fed mice and CD‐fed mice. (E) The F4/80 fluorescence in the livers of HFD‐fed mice and CD‐fed mice. (G) (H) The expression levels of M1‐type and M2‐type macrophage markers in bone marrow macrophages (BMMs) from HFD‐fed mice and CD‐fed mice. (I) The expression levels of M1‐type and M2‐type macrophage markers in the livers of HFD‐fed mice and CD‐fed mice. N = 5 in (A),(C), (D), (F) and (I), N = 4 in (G) and (H). *p < 0.05; **p < 0.01; ***p < 0.001

Compared with chow diet (CD)‐fed mice, the weight of HFD‐fed mice was significantly higher (Figure 1A). Oil Red O and H&E staining revealed a significant increase in hepatic lipid deposition in the livers of the HFD‐fed mice (Figure 1B). Notably, the phosphorylation of AKT and GSK was reduced in the livers of HFD‐fed mice, indicating impaired activation of the AKT/GSK pathway (Figure 1C). Moreover, the high‐fat diet also reduced glycogen synthesis in the livers of mice (Figure 1D). These data indicated that HFD‐fed mice developed obesity and insulin resistance. Additionally, the results of the immunofluorescence assay and western blot showed that macrophage infiltration was significantly increased in the livers of HFD‐fed mice (Figure 1E,F). BMMs from HFD‐fed mice were polarized towards an M1 activation profile, which was characterized by relatively high expression of *Tnf*‐*α*, *Il*‐*6* and *Mcp*‐*1*(Figure 1G) and low expression of *Cd206*, *Il*‐*10* and Arginase‐1(Figure 1H). Further, we also detected the polarization of macrophages in the liver of mice. Our results showed that compared with CD‐fed mice, macrophages in the liver of HFD‐fed mice were polarized to M1 with the high expression of *Tnf*‐*α*, *Il*‐*6* and *Mcp*‐*1*, but no change of M2 marker *Cd206*, and *Il*‐*10* and low expression of Arginase‐1 (Figure 1I).

### 3.2 Supernatants of BMMs from HFD‐fed mice and O/P‐treated RAW264.7 cells induced insulin resistance in Hep1‐6 cells

**FIGURE 2:**
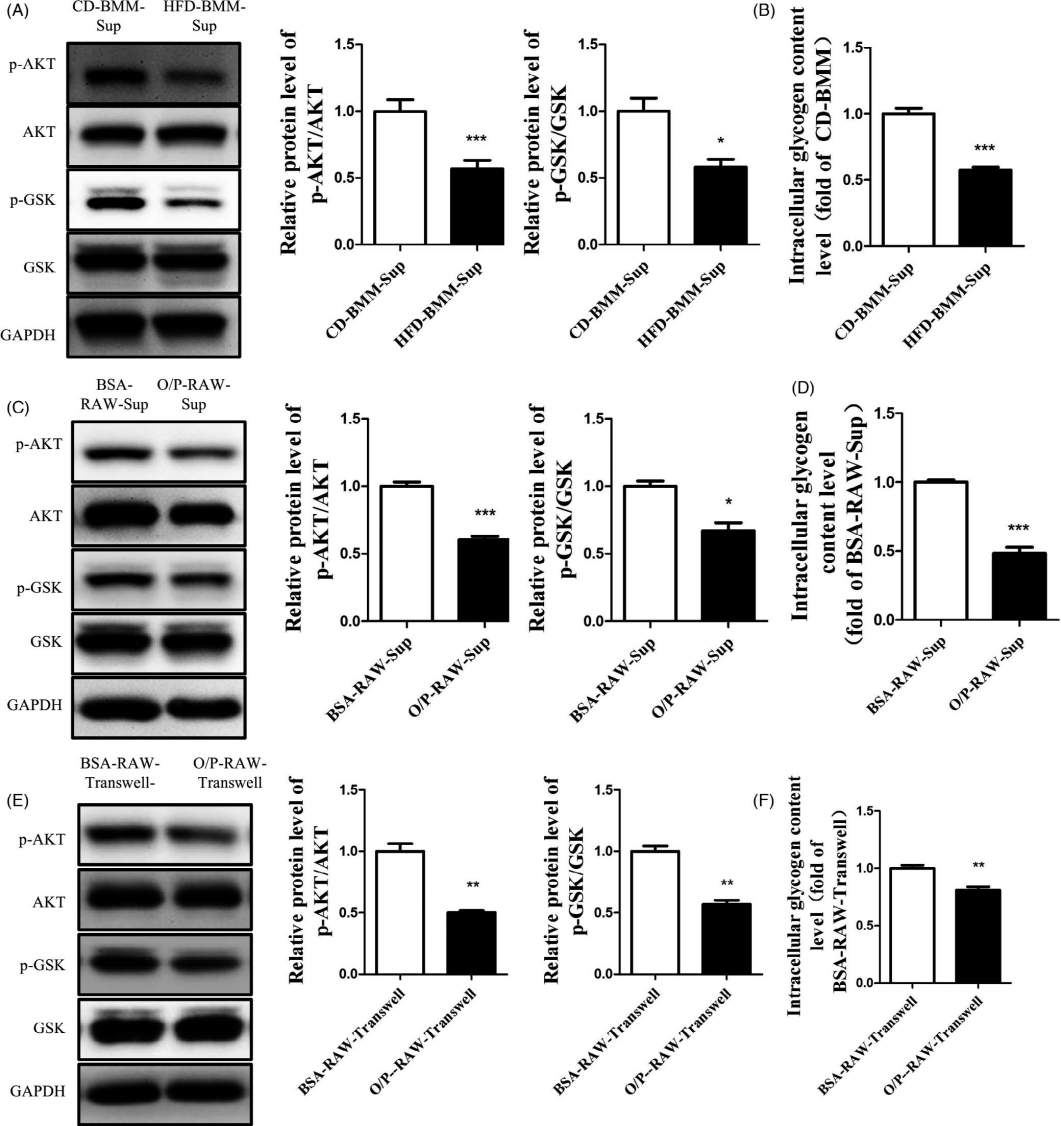
Supernatants of BMMs in HFD‐fed mice and O/P‐treated RAW264.7 cells induced insulin resistance in Hep1‐6 cells. BMMs from HFD‐fed mice and CD‐fed mice were obtained. The macrophage cell line RAW264.7 was treated with oleic acid/palmitic acid (O/P 2:1). (A)(B) The Hep1‐6 cells were treated with the supernatant from BMMs in HFD‐fed mice (HFD‐BMM‐Sup) and CD‐fed mice (CD‐BMM‐Sup). (C)(D) The Hep1‐6 cells were treated with the supernatant from RAW264.7 cells (O/P‐RAW‐Sup; BSA‐RAW‐Sup as control). (E)(F) Then Hep1‐6 cells were co‐cultured with O/P treated RAW264.7(O/P‐RAW‐Transwell) and BSA treated RAW264.7 (BSA‐RAW‐Transwell). The phosphorylation levels of AKT and GSK (A, C and E) and glycogen synthesis (B, D and F) in Hep1‐6 cells were measured. N=3 in (A) and (C)‐(F). N = 5 in (B). *p < 0.05; **p < 0.01; ***p < 0.001

To verify the relationship between the BMMs in HFD‐fed mice and liver cells, we obtained BMMs from HFD‐fed mice and CD‐fed mice. We collected the cell supernatant and centrifuged it to remove the cell debris. As shown in Figure 2A, compared with the BMMs from CD‐fed mice, the supernatant of BMMs from HFD‐fed mice impaired the activation of AKT and GSK, accompanied by decreased glycogen synthesis in Hep1‐6 cells (Figure 2B). Moreover, oleic acid/palmitic acid (O/P 2:1) was used to treat RAW264.7 cells to simulate BMMs from HFD‐fed mice. The results showed that the supernatant of the O/P‐treated RAW264.7 cells impaired the activation of AKT and GSK and lowered glycogen synthesis in Hep1‐6 cells (Figure 2C,D). Moreover, the O/P‐treated RAW264.7 and Hep1‐6 were co‐cultured using a transwell (membrane pore = 0.4 μm) plate. The activation of AKT and GSK were reduced in Hep1‐6 cells, accompanied by decreased glycogenesis (Figure 2E,F). These data indicated that the BMMs from HFD‐fed mice and O/P‐treated RAW264.7 cells secreted some factor that could induce hepatic insulin resistance.

### 3.3 Exosomes secreted by BMMs of HFD‐fed mice and O/P‐treated RAW264.7 cells mediated hepatocyte insulin resistance

**FIGURE 3:**
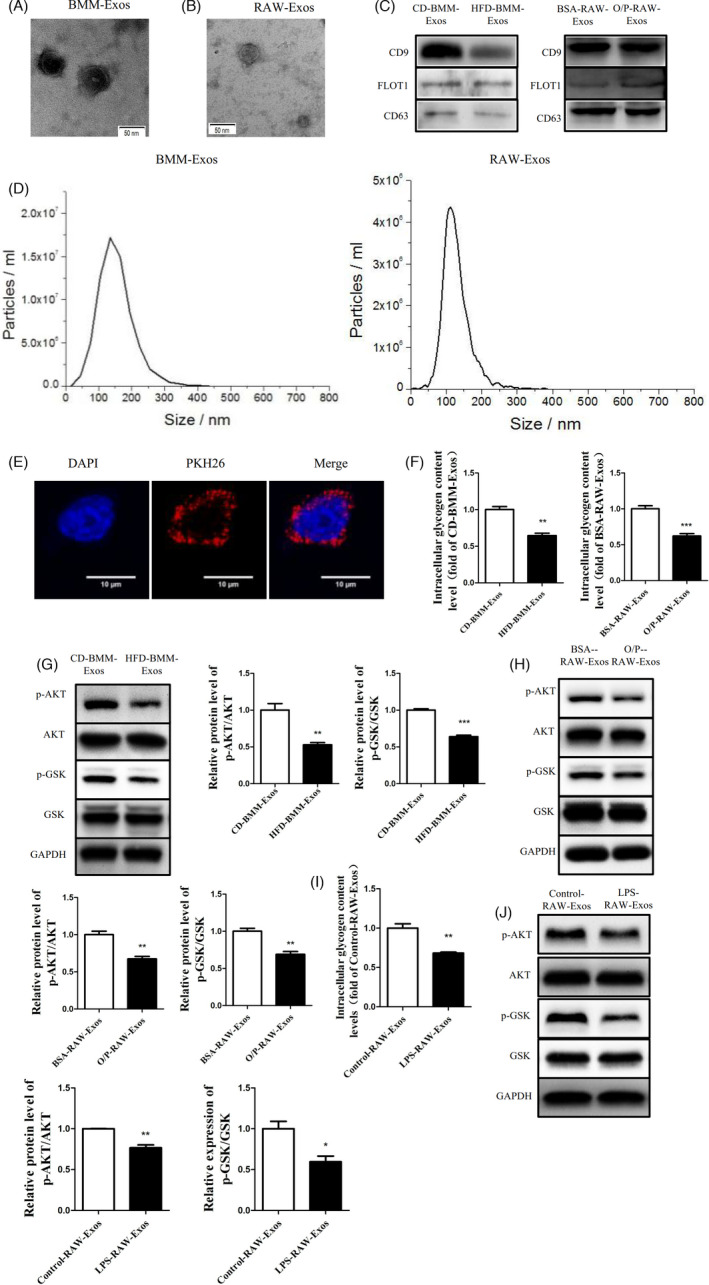
Exosomes secreted by the BMMs of HFD‐fed mice and the O/P‐treated RAW264.7 cells mediated hepatocyte insulin resistance. (A) The electron microscopy analysis of exosomes secreted by the BMMs of HFD‐fed mice (BMM‐Exos). (B) The electron microscopy analysis of exosomes secreted by the O/P‐treated RAW264.7 cells (RAW‐Exos). Scale bar, 50 nm. (C) The exosome‐specific CD9, FLOT1, and CD63 in CD‐BMM‐Exos, HFD‐BMM‐Exos, BSA‐RAW‐Exos and O/P‐RAW‐Exos were measured by western blot analysis. (D) The particle size of the exosomes secreted from BMMs and RAW264.7 cells were measured by NanoSight analysis. (E) The BMM‐Exos were labelled with PKH26 and then added to the Hep1‐6 cell cultures. (F) The level of glycogen synthesis in Hep1‐6 cells treated with CD/ HFD‐BMM‐Exos (*N* = 3) and BSA/O/P‐RAW‐Exos (*N* = 5). (G) (H) The phosphorylation levels of AKT/GSK in Hep1‐6 cells treated with CD/ HFD‐BMM‐Exos and BSA/O/P‐RAW‐Exos. (I) (J) The level of glycogen synthesis and phosphorylation levels of AKT/GSK in Hep1‐6 cells treated with Control‐RAW‐Exos and LPS‐RAW‐Exos. *N* = 3 in (G)‐(J). *p < 0.05; **p < 0.01; ***p < 0.001

Two types of cells that can interact via exosomes have been identified. Therefore, we speculated that exosomes secreted by the BMMs of HFD‐fed mice and O/P‐treated RAW264.7 cells could induce insulin resistance in liver cells. Both electron microscopy (EM) (Figure 3A,B), western blot (Figure 3C) and NanoSight analysis (Figure 3D) of isolated vesicles indicated that we successfully isolated exosomes using differential centrifugation. Next, these exosomes were labelled with the fluorescent dye PKH26 to test whether the exosomes could be taken up by Hep1‐6 cells. After adding labelled exosomes to the cell culture for 12 h, the Hep1‐6 cells exhibited efficient uptake of the exosomes, as indicated by the presence of red fluorescence staining in these cells (Figure 3E). Moreover, exosomes from both the BMMs of HFD‐fed mice and the O/P‐treated RAW264.7 cells reduced glycogen synthesis (Figure 3F) and phosphorylation of AKT and GSK (Figure 3G,H) in Hep1‐6 cells. To further reveal whether M1‐polarized BMMs could secrete exosomes to impair the insulin sensitivity of Hep1‐6 cells,the Hep1‐6 cells were treated with exosomes derived from LPS‐induced RAW264.7 (LPS‐RAW‐Exos). The result showed that LPS‐RAW‐Exos could impair the synthesis of glycogen and activation of AKT/GSK (Figure 3I,J). These data demonstrated that exosomes secreted by the BMMs of HFD‐fed mice and the O/P‐treated RAW264.7 cells could induce insulin resistance in liver cells.

### 3.4 Exosomes secreted by the BMMs of HFD‐fed mice induced insulin resistance in Hep1‐6 cells by delivering miR‐143‐5p

**FIGURE 4:**
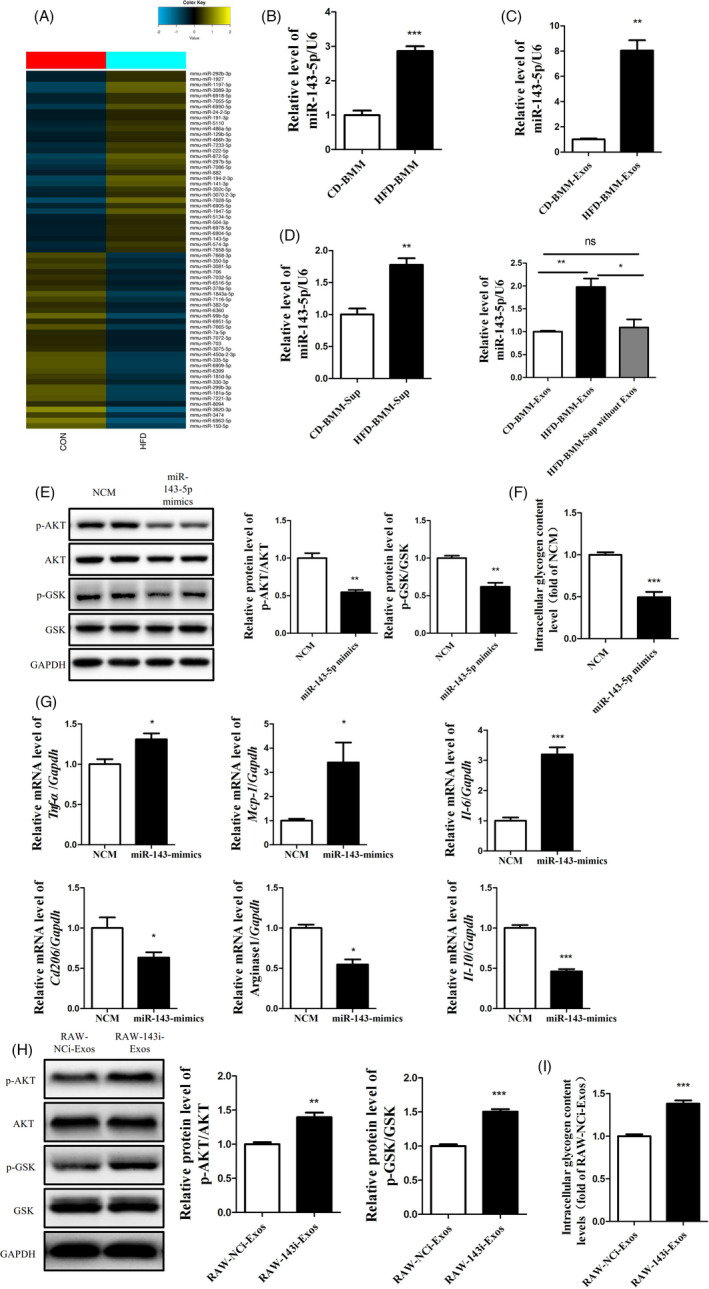
The exosomes secreted by the BMMs of HFD‐fed mice induced insulin resistance of Hep1‐6 cells by delivering miR‐143‐5p. (A) The differential expression levels of exosomal miRNAs in the CD‐BMM‐Exos and HFD‐BMM‐Exos. (B) The expression levels of miR‐143‐5p in the CD‐BMM and HFD‐BMM. (C) The levels of miR‐143‐5p in the CD‐BMM‐Exos and HFD‐BMM‐Exos. (D) The expression levels of miR‐143‐5p in Hep1‐6 cells treated with the supernatant and exosomes from the CD‐BMM and HFD‐BMM. (E) The activation of the AKT/GSK pathway and glycogen content (F) in Hep1‐6 cells treated with miR‐143‐5p mimics for 48 h. (G) The expression levels of M1‐type and M2‐type macrophage markers in the Hep1‐6 cells treated with miR‐143‐5p mimics for 48 h. The activation of the AKT/GSK pathway (H) and glycogen content (I) in Hep1‐6 cells treated with exosomes derived from RAW264.7 cells treated with miR‐143‐5p inhibitors (RAW‐143i‐Exos) for 48 h. *N* = 3 in (B)‐(E) and (H)‐(I). *N* = 4 in (F) (G). *p < 0.05; **p < 0.01; ***p < 0.001

It has been reported that exosomes could affect the biological function of recipient cells by delivering microRNAs. Therefore, a microRNA array was performed to detect the levels of microRNAs in the exosomes secreted by the BMMs of HFD‐fed mice and CD‐fed mice. The results showed 32 increased microRNAs in the exosomes secreted by the BMMs of HFD‐fed mice compared with the BMMs of CD‐fed mice (Figure 4A). To confirm the array results, the changes in upregulated microRNAs were examined using a real‐time PCR. As shown in Figure 4B,C, miR‐143‐5p was significantly upregulated in the BMMs of HFD‐fed mice and their exosomes. The level of miR‐143‐5p was much higher in macrophages compared with hepatocytes (Approximately 15 times)(Figure S1). Moreover, we found increased miR‐143‐5p in Hep1‐6 cells treated with the supernatant and exosomes from the BMMs of HFD‐fed mice (Figure 4D). Next, to investigate the role of miR‐143‐5p in hepatic insulin resistance, miR‐143‐5p mimics were transfected into Hep1‐6 cells. The overexpression of miR‐143‐5p in Hep1‐6 cells led to decreased phosphorylation of AKT and GSK and reduced glycogen synthesis (Figure 4E,F). Moreover, RAW264.7 cells transferred to M1 polarization after transfected with miR‐143‐5p mimics for 48h (Figure 4G). And exosomes derived from miR‐143‐5p‐inhibited RAW264.7 cells couldn't impair the activation of AKT/GSK and the synthesis of glycogen in Hep1‐6 (Figure 4H,I). Together, these data suggested that the exosomes secreted by the BMMs of HFD‐fed mice delivered miR‐143‐5p into Hep1‐6 cells and induced insulin resistance in Hep1‐6 cells.

### 3.5 *Mkp5* was identified as a target of miR‐143‐5p

**FIGURE 5:**
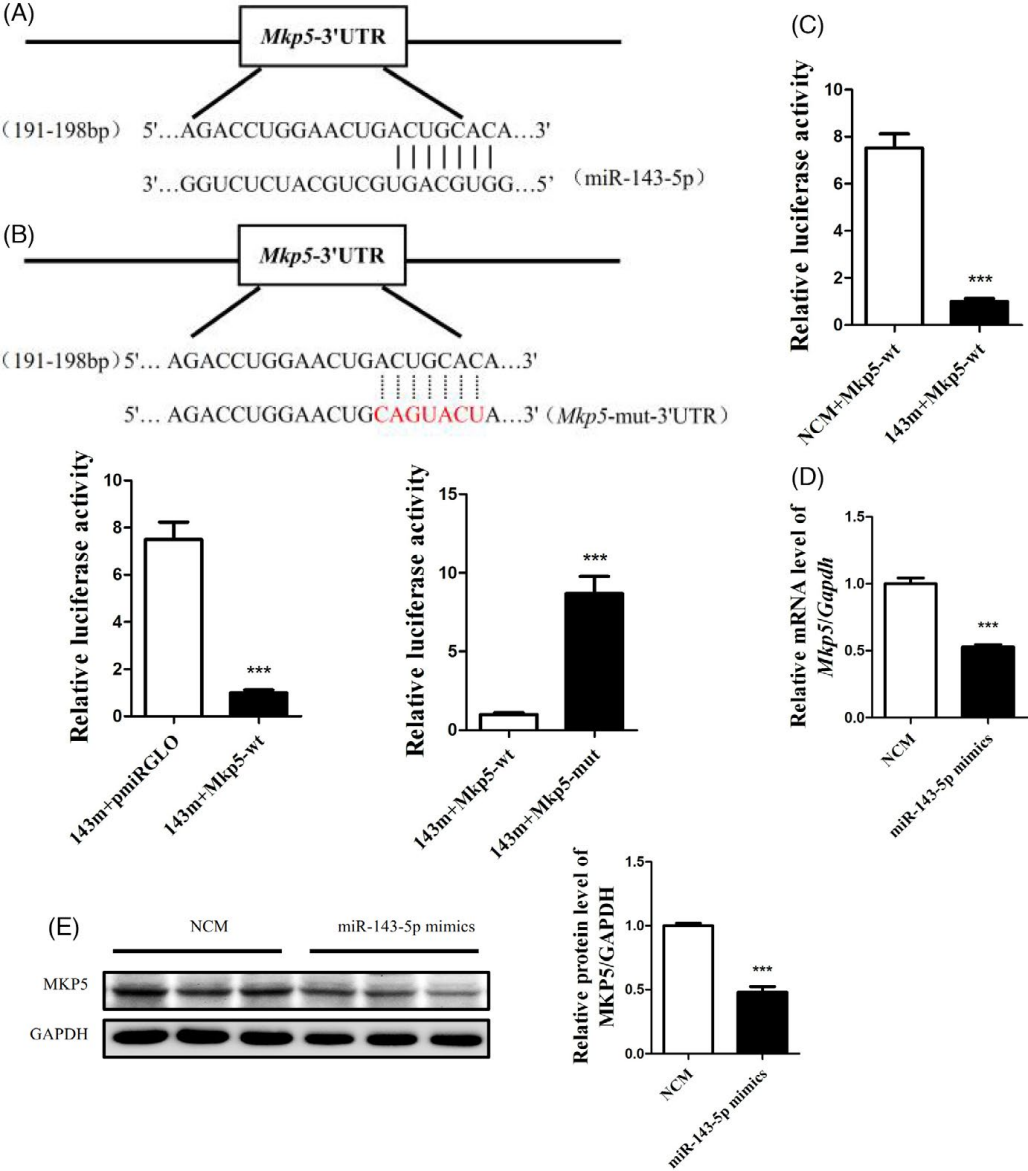
*Mkp5* was identified as a target of miR‐143‐5p. (A) The analysis of the target of miR‐143‐5p by the miRNA target prediction database TargetScan. (B) The mutation site of the 3′UTR of *Mkp5*. (C) The reduced luciferase activity in Hep1‐6 cells transfected with miR‐143‐5p mimics and the luciferase reporter vector containing the 3′UTR of *Mkp5*. (D) The mRNA level of *Mkp5* in the Hep1‐6 cells transfected with the miR‐143‐5p mimics. (E) The protein level of MKP5 in the Hep1‐6 cells transfected with the miR‐143‐5p mimics. *N* = 6 in (C). *N* = 3 in (D) and (E). *p < 0.01; ***p < 0.001

We next identified the potential target of miR‐143‐5p involved in glycogen synthesis using computational miRNA target prediction databases. The results from TargetScan revealed that there is a binding site of miR‐143‐5p on the 3′UTR of *Mkp5* (Figure 5A). To further verify that *Mkp5* is the target of miR‐143‐5p, we cloned the 3′UTR of *Mkp5* containing the predicted binding site and its mutation site (Figure 5B) into the pmirGLO vector. As shown in Figure 5C, transfection with the miR‐143‐5p mimics significantly impaired the luciferase activity in 293T cells transfected with the luciferase reporter vector containing the 3′‐UTR of *Mkp5* compared with those transfected with the miRNA mimic control. Similarly, the overexpression of miR‐143‐5p significantly reduced luciferase activity in 293T cells being transfected with pmirGLO‐*Mkp5*‐3′UTR compared with the pmirGLO vector and the pmirGLO‐Mkp5‐mutation‐3′UTR, indicating that *Mkp5* is a target of miR‐143‐5p. Moreover, the mRNA and protein levels of *Mkp5* were decreased in Hep1‐6 cells transfected with miR‐143‐5p mimics (Figure 5D,E). These results suggested that *Mkp5* is a target of miRNA‐143‐5p.

### 3.6 *Mkp5* participated in hepatic insulin resistance

**FIGURE 6:**
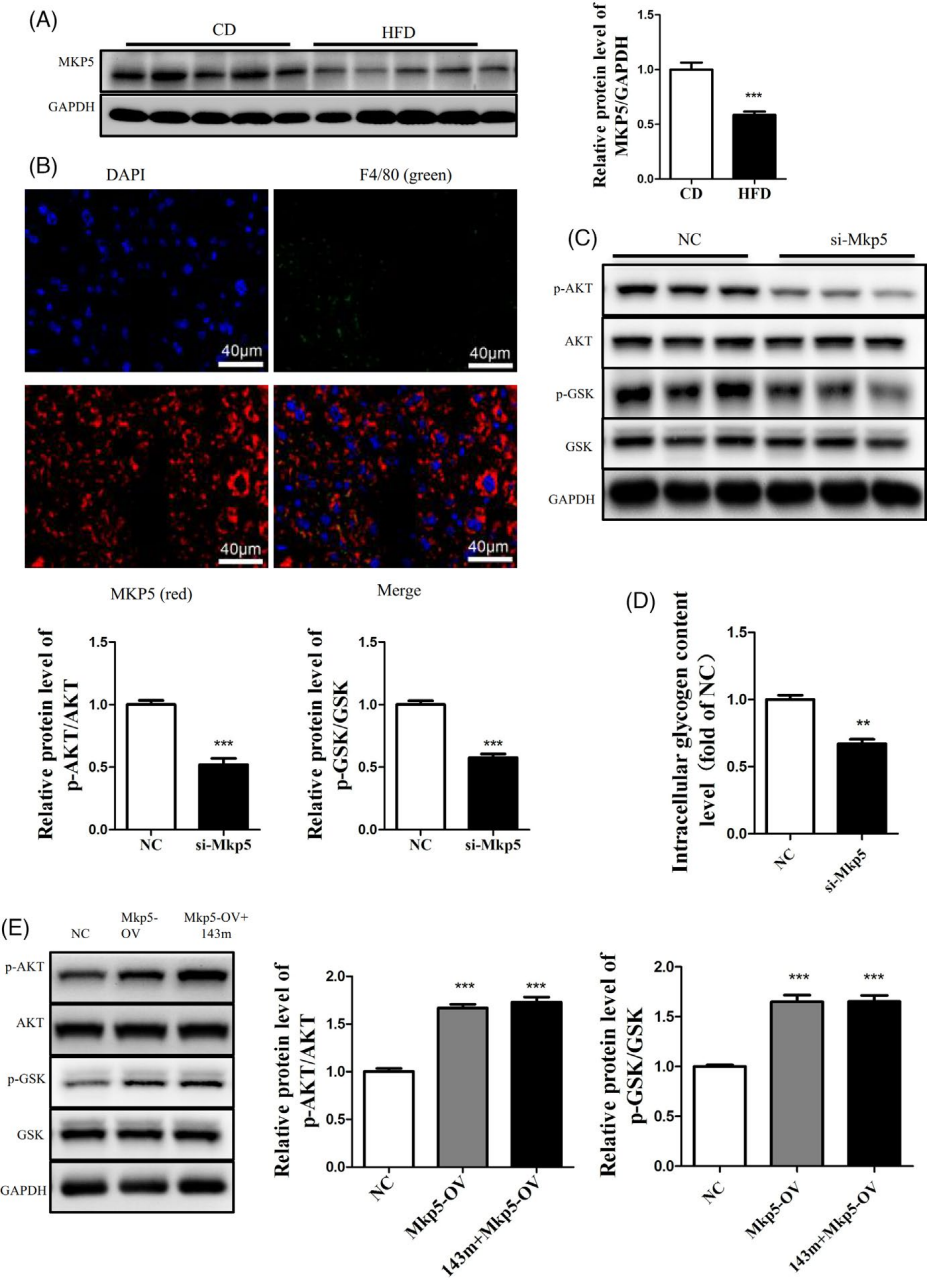
*Mkp5* participated in hepatic insulin resistance. (A) The protein level of MKP5 in the livers of HFD‐fed mice and CD‐fed mice. (B) The distribution of MKP5 in the liver of HFD‐fed mice, as shown by immunofluorescence assay. (C) The phosphorylation levels of AKT/GSK in Hep1‐6 cells transfected with si‐*Mkp5* for 48 h. (D) The glycogen content in Hep1‐6 cells transfected with si‐*Mkp5* for 48 h. (E) The phosphorylation levels of AKT/GSK in Hep1‐6 cells transfected with the miR‐143‐5p mimics and *Mkp5* Overexpression vector (*Mkp5*‐OV) for 48 h. *N* = 5 in (A). *N* = 3 in (C)(D). *N* = 4 in (E). *p < 0.05; **p < 0.01; ***p < 0.001

In this study, we found that the protein level of MKP5 was significantly decreased in the livers of HFD‐fed mice (Figure 6A). In addition, the immunofluorescence assay showed that the MKP5 was expressed in the hepatocytes and macrophages in the liver of HFD‐fed mice. (Figure 6B). To further explore the role of *Mkp5* in hepatic insulin resistance, we inhibited the expression of *Mkp5* in Hep1‐6 cells by transfection with siRNA targeting *Mkp5*. The transfection of si‐*Mkp5* reduced phosphorylation of AKT and GSK and glycogen synthesis in Hep1‐6 cells (Figure 6C,D). Furthermore, the overexpression of MKP5 reversed the effects of the miR‐143‐5p mimics on the phosphorylation levels of AKT/GSK (Figure 6E). Taken together, these findings indicated that miR‐143‐5p‐mediated insulin resistance by suppressing MKP5 expression.

## 4 DISCUSSION

In this study, we identified that in the liver of HFD‐fed mice, BMMs were polarized towards the M1 state, which induced hepatic insulin resistance by secreting exosomes.

Studies have found that macrophages play an important role in liver inflammatory fibrosis and chronic injury. In the injured liver, macrophages recruited from bone marrow could regulate liver inflammation and fibrosis.^23^ MCP‐1 is a classic chemokine that induces the migration of macrophages. The pharmacological inhibition of MCP‐1 or its receptor CCR2 could reduce the infiltration of BMMs in the liver and liver damage. Therefore, macrophages, especially bone marrow‐derived macrophages, have an important role in liver inflammation and are effective therapeutic targets during liver injury.^24^, ^25^ Different polarization of macrophages could produce different effects. Proinflammatory M1 macrophages promoted inflammation, and M2 macrophages suppressed inflammation. Obesity could induce adipose tissue macrophages to differentiate into the proinflammatory M1 type, which is related to the inflammation produced by adipose tissue and insulin resistance.^26^ Our results showed that in the livers of HFD‐fed mice, the activation of the insulin signalling pathway was impaired, glycogen synthesis was reduced, and infiltration of macrophages was significantly increased. The increased macrophages in the liver were recruited from bone marrow cells. Furthermore, compared with the BMMs of CD‐fed mice, the BMMs of HFD‐fed mice were obviously polarized towards M1 proinflammatory macrophages. This polarization further led to the appearance of inflammation in the body.

To determine the relationship between M1 BMMs and hepatocytes, we speculated that exosomes secreted by these BMMs might play an important role in hepatic insulin resistance. Exosomes are vesicles with a diameter of 30–100 nm secreted by cells or tissues^27^ that were first discovered by EG Trams in the supernatant of sheep erythrocytes cultured in vitro and given the name exosomes. Exosomes can mediate cell‐to‐cell communication by carrying a series of substances, such as nucleic acids, proteins, and cholesterol.^28^ In recent years, an increasing number of studies have indicated that exosomes can carry microRNAs into recipient cells and regulate the biological function of the recipient cells.^29^, ^30^ Studies have proven that exosomes derived from the adipose tissue of obese mice could modulate the insulin signalling pathway in hepatocytes by transferring miR‐141‐3p to inhibit the expression of PTEN.^31^ Similarly, the expression of miR‐29a was increased in adipose tissue‐derived exosomes of obese mice, which could be transferred into adipose tissue, the liver and skeletal muscle to regulate the insulin signalling pathway.^32^

To explore whether the exosomes secreted by the BMMs of HFD‐fed mice could carry microRNA to regulate insulin resistance in hepatocytes, exosomes derived from BMMs were collected in HFD‐fed mice and CD‐fed mice, and microRNA chip detection was used to screen the differentially expressed microRNAs. The real‐time PCR of the microRNAs showed that the miR‐143‐5p level in the exosomes secreted by the BMMs of HFD‐fed mice was significantly increased, which was consistent with the results of the microRNA array. miR‐143‐5p was also significantly increased in Hep1‐6 cells treated with exosomes secreted by the BMMs of HFD‐fed mice. It was reported that miR‐143‐5p played an important role in the regulation of gene expression. miR‐143‐5p could directly target a variety of mRNAs and participate in a series of processes, such as cell proliferation, differentiation and apoptosis. However, the role of miR‐143‐5p in hepatic insulin resistance is still unknown. Therefore, we speculated that miR‐143‐5p might play a role in hepatic insulin resistance mediated by BMMs. We found that miR‐143‐5p could directly target the 3′UTR of *Mkp5*. *Mkp5* is a member of the dual‐specificity phosphatase family and a key negative regulator of the protein kinase MAPK.^33^, ^34^ Studies have indicated that *Mkp5* is abundantly expressed in human and mouse insulin‐related tissues and organs such as the liver and skeletal muscle.^35^, ^36^ *Mkp5* is necessary for the obesity‐induced insulin response and can prevent the development of insulin resistance and metabolic abnormalities.^37^ All of these results showed that *Mkp5* was closely related to the occurrence and development of insulin resistance. In the present study, a luciferase reporter gene analysis proved that miR‐143‐5p could target *Mkp5* and significantly downregulate its expression. At the same time, it was found that the protein level of MKP5 was significantly downregulated in the livers of HFD‐fed mice. In vitro experiments showed that the overexpression of *Mkp5* reversed the damage to the insulin signalling pathway induced by the overexpression of miR‐143‐5p. Therefore, we proved that miR‐143‐5p inhibited MKP5 expression to regulate the insulin signalling pathway in hepatocytes.

In summary, our study confirmed that obesity in HFD‐fed mice could cause BMMs to polarize to the M1 type and secrete exosomes to deliver miR‐143‐5p into hepatocytes. miR‐143‐5p subsequently regulated the insulin signalling pathway in hepatocytes by suppressing MKP5 expression.

## CONFLICT OF INTEREST

The authors declare no conflict or financial interest.

## AUTHOR CONTRIBUTIONS

Linfang Li conducted the experiments, analysed the results and wrote the paper. Huiyan Zuo conducted experiments on the animal model. Xiuqing Huang, Tao Shen Xiaoyi Zhang, Tong An and Weiqing Tang conducted the isolation of exosomes. Lin Dou and Jian Li conceived the idea for the project and revised the manuscript. All authors reviewed and approved the final manuscript.

## Supporting information

Figure S1

## Data Availability

All data included in this study are available upon request by contacting the corresponding author.
