# Supplementary figures and images for: Bone marrow macrophage‐derived exosomal miR‐143‐5p contributes to insulin resistance in hepatocytes by repressing MKP5
Source: Cell Prolif. 2021 Oct 14;54(12):e13140. doi: 10.1111/cpr.13140 (PMC8666281; doi:10.1111/cpr.13140)

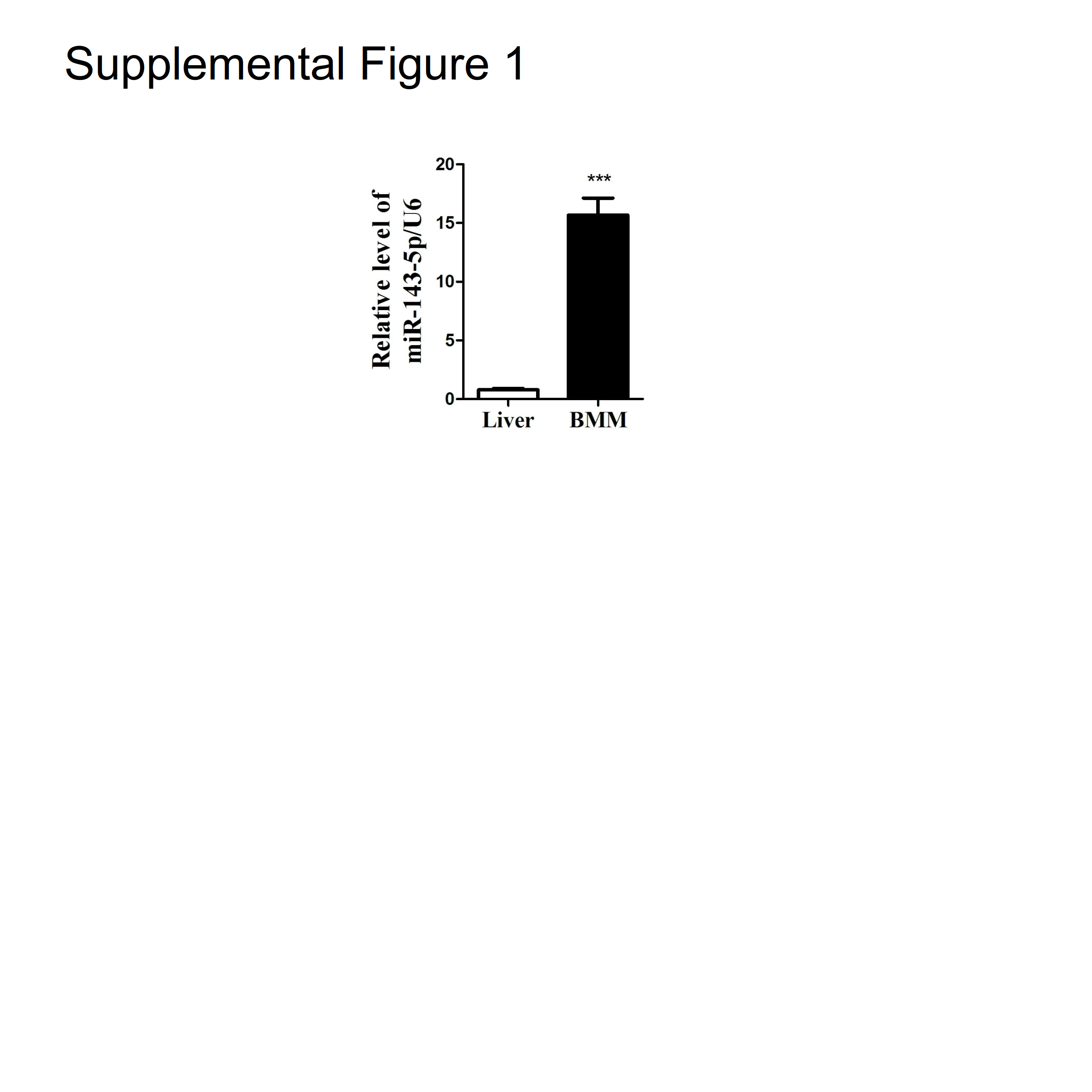

Supplement: Supplementary file 1 — Figure S1 [file CPR-54-e13140-s001.tif]
